# Drug related problems in type 2 diabetes patients with hypertension: a cross-sectional retrospective study

**DOI:** 10.1186/1472-6823-13-2

**Published:** 2013-01-07

**Authors:** Hasniza Zaman Huri, Hoo Fun Wee

**Affiliations:** 1 Department of Pharmacy, Faculty of Medicine, University of Malaya, Kuala Lumpur, 50603, Malaysia

**Keywords:** Type 2 diabetes, Hypertension, Drug-related problems

## Abstract

**Background:**

Type 2 diabetes (T2DM) patients with hypertension are at increased risk for experiencing drug-related problems (DRPs) since they often receive multiple medications and have multiple comorbidities. To date, there is a lack of studies conducted in T2DM patients with hypertension. This study aims to analyze the DRPs and identify factors affecting the DRPs in this patient population.

**Method:**

This retrospective study involved T2DM patients with hypertension and was conducted at a tertiary hospital in Malaysia from January 2009 to December 2011. The assessment of DRPs was based on the Pharmaceutical Network Care Europe (PCNE) tool version 5.01.

**Results:**

Two hundred patients with a total of 387 DRPs were identified. Among these patients, 90.5% had at least one DRP, averaging 1.9 ± 1.2 problems per patient. The most common DRPs encountered were insufficient awareness of health and diseases (26%), drug choice problems (23%), dosing problems (16%) and drug interactions (16%). The most implicated drugs were aspirin, clopidogrel, simvastatin, amlodipine and metformin. The six domains of DRPs found to have statistically significant associations were renal impairment, polypharmacy, cardiovascular disease, elderly status, and duration of hospital stay.

**Conclusions:**

Early identification of the types and patterns of DRPs and the factors associated to them may enhance the prevention and management of DRPs in T2DM patients with hypertension.

## Background

Malaysia is one of the top ten countries in the world with the greatest number of diabetes patients [1] and the prevalence has increased dramatically from 8.3% in 1996 to 14.9% in 2006, affecting 1.4 million adults aged ≥ 30 years old [2]. According to the results of DiabCare Malaysia 2008, Type 2 diabetes (T2DM) accounts for more than 90% of all cases in adults [3]. Hypertension is a common comorbidity in T2DM patients, with a prevalence of up to two-thirds of the population, and it may be present by the time T2DM is diagnosed or even before the onset of hyperglycemia [4]. Hypertension enhances the risk of cardiovascular disease in T2DM patients [4]. It also increases the risk of developing microvascular complications such as diabetic nephropathy and retinopathy [5,6].

To minimize the risk of complications, many guidelines recommend a target blood pressure (BP) of ≤ 130/80 mmHg in all T2DM patients with hypertension [4,7,8]. However, achieving this target BP remains a great challenge and the majority of the patients require one or more antihypertensive agents in order to achieve this optimal BP control [9].

T2DM patients with hypertension often receive multiple medications and this can lead to the occurrence of drug-related problems (DRPs) [9]. A high prevalence of DRPs has been observed in T2DM patients [10,11]. DRPs may lead to suboptimal blood pressure control [12] which can contribute to significant morbidity or mortality, prolonged hospitalization, and increased health care expenditure if left unresolved [13]. However, in most cases, these DRPs are preventable [14].

There are several factors influencing DRPs in T2DM patients with hypertension. Polypharmacy (≥ 5 concurrent medications) is an inherent factor as high blood pressure and diabetic complications usually complicate the treatment [9]. Age status (≥ 65 years old) is another factor, given increased association with multiple medical conditions, multiple drug therapies and age-related changes in the pharmacokinetics and pharmacodynamics of drugs [9]. Multiple medical conditions and renal impairment also have been shown to contribute to DRPs [15,16].

To date, there has been a lack of studies conducted locally and globally to investigate and document DRPs in T2DM patients with hypertension. Our study’s aim was to provide baseline data regarding DRPs to allow the implementation of more effective management and to reduce the mortality and morbidity associated with DRPs.

### Objectives

1. To assess the drug-related problems in type 2 diabetes patients with hypertension.

2. To identify the factors influencing drug-related problems in type 2 diabetes patients with hypertension.

## Methods

### Study design and setting

This was a retrospective study conducted in Malaysia’s premier teaching hospital with 1000-beds, the University of Malaya Medical Centre (UMMC).

### Study population

**Figure 1:**
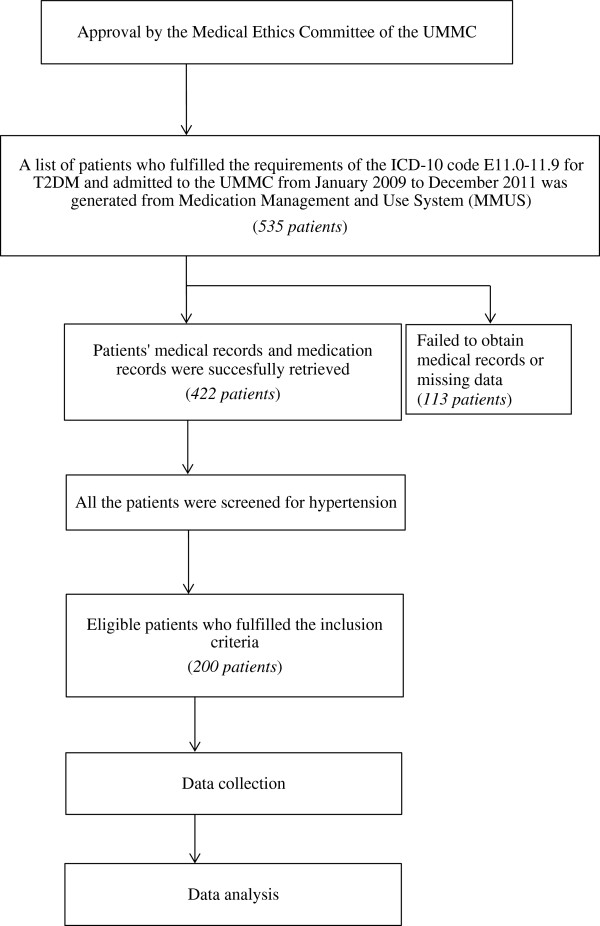
Overview of study procedure.

A total of 200 patients were included in this study. The sample size was calculated using the Epi Info, Version 6 (Centers for Disease Control and Prevention, Atlanta GA), which provided a minimum sample size of 195 patients. The study population consisted of T2DM patients with hypertension who fulfilled the requirements of the International Classification of Diseases Tenth Revision (ICD-10) code E11.0-11.9 for T2DM and who were admitted to the UMMC from January 2009 to December 2011. The study complied with the Declaration of Helsinki and the ethics committee of UMMC granted its approval. An overview of the study procedure is shown in Figure 1.

#### Inclusion criteria

1. Patients diagnosed with T2DM and hypertension

2. Patients who received at least one antidiabetic drug (oral antidiabetic drug or insulin) and at least one antihypertensive agent in the ward

3. Adult patients aged 18 years old and above

#### Exclusion criteria

1. Patient with missing data

### Data collection

Data were collected by the authors who are pharmacists. Demographic characteristics such as age, gender, ethnicity, height, weight, and body mass index were recorded. Clinical characteristics such as duration of hospital stay, duration of T2DM, duration of hypertension, presence of diabetic complications (referred to diabetic retinopathy, neuropathy or diabetic foot ulcer), presence of comorbidities, laboratory results and concurrent medications were also collected.

#### Definition used in the study

● Comorbidities were defined as chronic illnesses or diseases which require long term treatment [17].

● Cardiovascular events referred to the presence of acute coronary syndromes, ischemic heart disease, heart failure, arrhythmias, cardiomyopathy or as stated in the medical records [18].

● Renal impairment was defined as chronic kidney disease, chronic interstitial nephritis, chronic glomerulonephritis, creatinine clearance of less than 35 mL/min, diabetic nephropathy, nephrosclerosis or as stated in the medical records [19]. The assessment of the creatinine clearance with the use of drugs was based on BNF and Lexicomp.

● Liver impairment referred to liver cirrhosis, chronic hepatitis, hepatocellular carcinoma, elevations of liver enzymes (more than 3 times the upper normal limits) or as stated in the medical records [19,20].

● Polypharmacy was defined as the use of five or more medications [21].

● DRP is defined as “an event or circumstance involving drug therapy that actually or potentially interferes with desired health outcomes” [22].

● Significant potential drug interactions are defined as interactions that potentially can cause harm to patient and are well documented or can cause moderate harm without well documented studies [23].

Note: The same drug used with different strengths was counted as one item whereas the same drug with different routes of administration was counted as separate items. Combinations of drugs were counted as a single item. However, this did not apply to antidiabetic and antihypertensive drugs, where the number of drugs used was counted according to the number of classes.

### Classification, identification and assessment of DRPs

The Pharmaceutical Care Network Europe (PCNE) classification of DRPs version 5.01 [22] was used to categorize DRPs. It is an established system that has been revised several times and its validity and reproducibility have been tested [11,24]. It has been used by many recent studies [11,21,25].

In this study, the six domains of problems of the PCNE classification were used. The DRPs and their possible causes were identified from the patients’ medical records, with reference to the standard guidelines and established literatures [4,7,8,26,27]. Two main references were used to assess the appropriateness of drug indications, appropriateness of drug and dosage, possible drug interactions, adverse drug reactions and contraindications [28,29]. The authors who are pharmacists were involved in the identification and classification of DRPs.

#### Modified Beers criteria

The modified Beers criteria [30,31] were used in this study. This is a consensus-based drug list that includes a number of drugs which should be avoided or used very cautiously in the elderly. For this study, the criteria were used as a reference to assess and identify the potential drugs that were inappropriately prescribed in the T2DM patients with hypertension who were aged 65 and above. The listed drugs were generally divided into low and high risk. In this study, only inappropriate prescriptions of “Beers criteria high severity” drugs were identified as DRPs because these drugs might pose clinically significant adverse effects when used in the elderly.

### Statistical Techniques

The statistical software Statistical package for Social Science (SPSS) version 19 (SPSS Inc., Chicago, IL, USA) was used to analyze all the data collected and extracted in this study. Categorical data were expressed as percentages and continuous data were expressed as mean ± standard deviation. Independent Student’s *t-*tests were used to compare group means of continuous dependent variables while the Kolmogorov-Smirnov Test of Normality were used to test the distribution of a sample.

In addition, the association or correlation between two categorical variables was examined using the chi-square test of independence. Statistical significance was defined as *p*-value < 0.05. Refer to Figure 1.

## Results

A total of 200 patients were included in this study. The mean age of patient was 62.3 ± 12.7 years old and the non-elderly patients were 15.0% more numerous than the elderly patients. The minimum and maximum ages of the patients were 31 and 95 years old, respectively. Table 1 demonstrates the demographic and clinical characteristics of this study population. There was an average of 4.6 ± 1.3 chronic illnesses per patient. The mean number of medications was 6.9 ± 2.8. The number of medications taken by patients ranged from 3 to 20. Polypharmacy was common as 76.5% of the patients were taking five or more medications, of whom 46.4% were elderly patients.

**Table 1:** Demographic and clinical characteristic of the patients (N = 200)

**Characteristics**	**Number of patients (Percentage, %)**
**Sex**	
Male	103(51.5)
Female	97 (48.5)
**Age**	
Non-elderly	115 (57.5)
Elderly	85 (42.5)
**Duration of hospital stay**	
Not more than 7 days	143 (71.5)
8 to 14 days	38 (19.0)
More than 15 days	19 (9.5)
**Duration of type 2 diabetes mellitus**	
Not more than 10 years	72 (36.0)
11 to 20 years	47 (23.5)
21 to 30 years	31 (15.5)
Unknown duration	50 (25.0)
**Duration of hypertension**	
Not more than 10 years	82 (41.0)
11 to 20 years	43 (21.5)
21 to 30 years	22 (11.0)
Unknown duration	53 (26.5)
**A1c**	
Achieved target (< 6.5)	48 (24.0)
Did not achieve target (≥ 6.5)	146 (73.0)
Unknown	6 (3.0)
Diabetic complications*	
Diabetic retinopathy	43 (21.5)
Diabetic foot ulcer	21 (10.5)
Diabetic neuropathy	17 (8.5)
**Comorbidities**^**†**^	
Renal impairment	100 (50.0)
Cardiovascular disease	96 (48.0)
Dyslipidemia	64 (32.0)
Stroke	40 (20.0)
Gastrointestinal disease	14 (7.0)
Liver impairment	11 (5.5)
Bronchial asthma	7 (3.5)
Benign prostatic hyperplasia	7 (3.5)
Gouty arthritis	6 (3.0)
Osteoarthritis	5 (2.5)

^†^One patient may have more than one diabetic complication or comorbidity.

Patients on combination therapy were 4.5% more numerous than patients on monotherapy, and the most frequently used drug classes were calcium channel blockers, ACE inhibitors and diuretics. Amlodipine, perindopril and frusemide were the most widely used agents representing these classes, respectively.

The dual therapy that had the highest frequency was ACE inhibitors plus calcium channel blockers (5%), followed by ACE inhibitors plus beta blockers (4.5%). The combination of ACE inhibitors, calcium channel blockers and beta blockers (4%) was the most commonly used triple therapy. Additionally, 2 out of the 19 patients received antihypertensive agents from 5 different classes.

More than two-thirds of the patients were on monotherapy with oral antidiabetic agents or insulin. Insulin was more widely used than oral agents, with a difference of 17.5%. For combination therapy, oral agents plus insulin (17.5%) appeared to be the most common combination. Of these, insulin plus a single antidiabetic agent had the highest frequency (14%).

Insulin was the most common antidiabetic prescribed. The most frequently used oral agents were biguanides (metformin) and sulfonylureas. The use of acarbose (alpha-glucosidase inhibitors) and sitagliptin (dipeptidyl peptidase-4 inhibitors) were 3.2% and 0.6%, respectively.

A total number of 387 DRPs were identified (Table 2). There was an average of 1.9 ± 1.2 problems and 1.7 ± 1.1 causes per patient. A total of 90.5% of the patients had at least one DRP. The most frequent categories of DRPs were “others”, drug choice problems and drug interactions. The mean number of chronic illnesses was 2.65 ± 1.3 in the group of patients with DRPs and 2.1 ± 1.1 in the group of patient without DRPs. There was a significant difference (p = 0.039) between the two group when tested using the independent sample *t*-test. Under the “others” category, the most frequent problem encountered was the “insufficient awareness of health and diseases,” accounting for 22% of all the cases. On the other hand, most of the drug choice problems were the results of inappropriate drug selection and the use of contraindicated drugs.

**Table 2:** Drug related problems in type 2 diabetes patients with hypertension (n = 387)

**Code**	Problems*	**Number of problem (Percentage, %)**
**P1**	**Adverse reactions**	**25 (6.5)**
P1.1	Side effects suffered (non-allergic)	25 (6.5)
**P2**	**Drug choice problems**	**87 (22.5)**
P2.1	Inappropriate drug (not most appropriate for indication)	34 (8.8)
P2.2	Inappropriate drug form (not most appropriate for indication)	4 (1.0)
P2.3	Inappropriate duplication of therapeutic group or active ingredient	4 (1.0)
P2.4	Contraindication for drug	29 (7.5)
P2.5	No clear indication for drug use	1 (0.3)
P2.6	No drug but clear indication	15 (3.9)
**P3**	**Dosing problems**	**62 (16.0)**
P3.1	Drug dose too low or dosage regime not frequent enough	5 (1.3)
P3.2	Drug dose too high or dosage regime too frequent	44 (11.3)
P3.3	Duration of treatment too short	8 (2.1)
P3.4	Duration of treatment too long	5 (1.3)
**P4**	**Drug use problems**	**50 (12.9)**
P4.1	Drug not taken/administered at all	50 (12.9)
**P5**	**Interactions**	**63 (16.3)**
P5.1	Potential interaction	63 (16.3)
**P6**	**Others**	**100 (25.8)**
P6.1	Patient dissatisfied with therapy despite taking drug(s) correctly	5 (1.3)
P6.2	Insufficient awareness of health and diseases (possibly leading to future problems)	90 (23.2)
P6.4	Therapy failure (reason unknown)	5 (1.3)

*Only problems that have a frequency of more than one were included.

A total of 25 adverse reactions were reported (Table 2). Antidiabetic drugs were associated with about one-third of all the cases. Eight patients experienced hypoglycemia secondary to either oral antidiabetic drugs or insulin. Tremor secondary to insulin was also recorded. Antihypertensive agents that caused adverse reactions were calcium channel blockers, diuretics and ACE inhibitors. It was reported that amlopidine had caused increased heart rate and bilateral leg swelling. Electrolyte imbalances were reported as adverse reactions of perindopril (hyperkalemia) and indapamide (hyponatremia). Also, urinary hesitancy secondary to hydrochlorothiazide had been reported. One patient suffered from rhabdomyolysis secondary to a lipid lowering agent. Drugs that did not fall into the above three classes were classified as “others”. For example, bleeding gums and hematuria secondary to warfarin had been reported. Also, steroids had been associated with two cases of adverse reactions. Among the 87 drug choice problems identified, most of the problems were related to inappropriate drug choice and contraindications. The distribution of the drug choice problem is displayed in Table 2. Antihypertensive drugs were not used appropriately. The inappropriate choice of antihypertensive drugs resulted in 19 drug choice problems. For example, ACE inhibitors were used in five patients with ESRD. Alpha blockers such as prazosin and doxazosin were used as second or third add-on therapies when other better alternatives were available and not contraindicated (7 patients). Short-acting nifedipine was used in two elderly patients and spironolactone was prescribed to two renal impairment patients with creatinine clearances of less than 30 mL/minute. Aspirin was also given to patients with severe renal impairment who had creatinine clearances of less than 10 mL/minute.

In addition to these, there were several drugs which were inappropriately used in the elderly and were classified as high risk under the modified Beers criteria, namely, ticlopidine (4 cases), amitriptyline (2 cases), diphenhydramine (2 cases), liquid paraffin (2 cases), chlorpheniramine, and others.

Twenty-eight drug choice problems involving the use of contraindicated drugs were identified. The three most common drugs that were most prescribed in the presence of contraindications were metformin, aspirin and fondaparinux. For instance, metformin was prescribed in 18 patients for whom it was contraindicated (e.g., creatinine clearance less than 30 mL/minute, more than 80 years old, or recent myocardial infarction). Also, gliclazide was prescribed to five patients with severe liver impairment and aspirin was given to patients who had a previous history of allergy. Three patients received fondaparinux despite their poor renal function (creatinine clearances of less than 30 mL/minute).

There were 15 cases where no drugs were prescribed for a clear indication. For example, hematinics were indicated in ESRD patients with chronic anemia but no drugs were prescribed (10 cases). Also, aspirin or simvastatin were not given to some of the patients as secondary prophylaxis of CVD.

Of the 62 cases of inappropriate dosing identified, most of the drugs were prescribed at a higher dose than required, particularly in patients with existing renal or liver impairments. The most common drugs that were involved in wrong dosages were H_2_-antogonists (20 cases), antibiotics (8 cases), antihypertensive agents (6 cases), antidiabetic drugs (3 cases) and others. Ranitidine was commonly prescribed at a higher dosage than required in patients with creatinine clearances of less than 50 mL/minute.

In addition to wrong dosages, inappropriate durations of treatment were also identified. Durations of treatment were too short in eight cases whereas longer than required in four cases. For example, oseltamivir (Tamiflu®) was only given for three days for the treatment of H1N1 and oral azithromycin 500 mg was administered once daily for six days.

One-quarter of the patients had at least one drug use problem and this made up approximately 13% of all the DRPs. It was recorded in the patients’ medical records that drugs were not taken or administered by them prior to admission. Non-compliance with antihypertensive agents and antidiabetic drugs was frequent.

There were a total of 63 significant potential drug interactions identified. The use of aspirin and clopidogrel posed a significant potential drug interaction in 25 patients. In addition, there were 14 patients who were prescribed simvastatin at more than 20 mg while receiving amlodipine. In this study, the drugs most implicated in drug interactions were aspirin (32 cases), clopidogrel (31 cases), simvastatin (23 cases), amlodipine (15 cases), omeprazole (15 cases), and iron salts (9 cases).

About one-quarter of the DRPs that could not be classified under any other category were regarded as “Others”. The majority of patients had insufficient awareness of health and diseases which could possibly lead to future problems. For example, many patients had a lack of knowledge about T2DM. Also, they were unaware of the management of the disease and its complications.

The total number of causes identified was 336 (Table 3). The causes varied with the problems identified. Some problems might have more than one cause whereas some might not have any causes. For example, most of the drug interactions did not have a cause. Of all the causes identified, inappropriate drug selection was the cause with the highest frequency, followed by inappropriate dosage selection and burden of therapy.

**Table 3:** Causes of DRPs in T2DM patients with hypertension (n = 336)

**Code**	Causes*	**Number of problem (Percentage, %)**
**C1**	**Drug/Dose selection**	**158 (47.0)**
C1.1	Inappropriate drug selection	70 (20.8)
C1.2	Inappropriate dosage selection	58 (17.2)
C1.5	Synergistic/preventive drug required and not given	15 (4.5)
C1.8	Manifest side effect, no other cause	15 (4.5)
**C2**	**Drug use process**	**36 (10.7)**
C2.1	Inappropriate timing of administration and/or dosing intervals	2 (0.6)
C2.2	Drug underused/under-administered	33 (9.8)
C2.6	Patient unable to use drug/form as directed	1 (0.3)
**C3**	**Information**	**13 (3.9)**
C3.1	Instructions for use/taking not known	1 (0.3)
C3.2	Patient unaware of reason for drug treatment	6 (1.8)
C3.4	Patient unable to understand local language	6 (1.8)
**C4**	**Patient/Psychological**	**124 (36.9)**
C4.1	Patient forgets to use/take drug	14 (4.2)
C4.2	Patient has concerns with drugs	4 (1.2)
C4.3	Patient suspects side-effect	10 (3.0)
C4.5	Patient unwilling to bother physician	6 (1.8)
C4.7	Patient unwilling to adapt life-style	28 (8.3)
C4.8	Burden of therapy	47 (14.0)
C4.9	Treatment not in line with health beliefs	15 (4.4)
**C5**	**Logistic**	**5 (1.5)**
C5.1	Prescribed drug not available (anymore)	3 (0.9)
C5.2	Prescribing error (only in case of slip of the pen)	2.(0.6)

*Only causes that have frequency of more than one were included.

When matching the cause to the problem, inappropriate drug or dose selection was found to be the most common cause for drug choice problems and dosing problems. The main cause for adverse reaction was “patient or psychological”, particularly “patient has concerns with drugs”. Drug use problems were commonly caused by “patient or psychological”, followed by “drug use process”. Similarly, “patient or psychological” was the most common cause for “other” problems.

There was no statistical significance identified when comparing factors with DRPs (Tables 4, 5). Despite this, several factors were found to have a statistically significant association with the 6 domains of DRPs. The elderly had a positive statistical association with drug choice problems (*p* < 0.001). Also, there were significant associations between renal impairment and drug choice problems (*p* = 0.029) or dosing problems (*p* = 0.027).

**Table 4:** Comparison between factors and occurrence of adverse reactions, drug choice problems, and dosing problems

**Factors**	**Adverse reactions (n = 25) Frequency (Percentage, %)**			**Drug choice problem (n = 76) Frequency (Percentage, %)**			**Dosing problem (n = 55) Frequency (Percentage, %)**		
	**Yes**	**No**	***p*-value**	**Yes**	**No**	***p*-value**	**Yes**	**No**	***p*-value**
**Elderly**									
Yes	11 (44.0)	74 (42.3)	> 0.999^a^	28 (36.8)	87 (70.2)	< 0.001^a^*	34 (61.8)	81 (70.4)	0.548^a^
No	14 (56.0)	101(57.7)		48 (63.2)	37 (29.8)		21 (38.2)	64 (29.6)	
**Polypharmacy**									
Yes	19 (76.0)	134 (76.6)	> 0.999^a^	60 (78.9)	93 (75.0)	0.640^a^	38 (69.1)	115 (79.3)	0.182^a^
No	6 (26.0)	41 (23.4)		16 (21.1)	31 (25.0)		17 (30.9)	30 (20.7)	
**Duration of hospital stay**									
≤ 1 week	17 (68.0)	126 (72.0)	0.859^a^	45 (59.2)	98 (79.0)	0.004^a^*	40 (72.7)	103 (71.0)	0.951^a^
> 1 week	8 (32.0)	49 (28.0)		31 (40.8)	26 (21.0)		15 (27.3)	42 (29.0)	
**Microvascular complications**									
Yes	17 (68.0)	107 (61.1)	0.660^a^	53 (69.7)	71 (57.3)	0.106^a^	40 (72.7)	84 (57.9)	0.078^a^
No	8 (32.0)	68 (38.9)		23 (30.3)	53 (42.7)		15 (27.3)	61 (42.1)	
**Cardiovascular events**									
Yes	12 (48.0)	109 (62.3)	0.193^b^	47 (61.8)	74 (59.7)	0.877^a^	36 (65.5)	85 (58.6)	0.471^a^
No	13 (52.0)	66 (37.7)		29 (38.2)	50 (40.3)		19 (34.5)	60 (41.4)	
**Renal impairment**									
Yes	12 (48.0)	88 (50.3)	> 0.999^a^	46 (60.5)	54 (43.5)	0.029^a^*	35 (63.6)	65 (44.8)	0.027^a^*
No	13 (52.0)	87 (49.7)		30 (39.5)	70 (56.5)		20 (36.4)	80 (55.2)	
**Liver impairment**									
Yes	1 (4.0)	10 (5.7)	> 0.999^b^	6 (7.9)	5 (4.0)	0.399^a^	5 (9.1)	6 (4.1)	0.306^a^
No	24 (96.0)	165 (94.3)		70 (92.1)	119 (96.0)		50 (90.9)	139 (95.9)	
**Hyperlipidemia**									
Yes	7 (28.0)	57 (32.6)	0.819^a^	17 (22.4)	47 (37.9)	0.033^a^*	15 (27.3)	49 (33.8)	0.476^a^
No	18 (72.0)	118 (67.4)		59 (77.6)	77 (62.1)		40 (72.7)	96 (66.2)	

^a^ Computed using Continuity Correction; ^b^ Computed using Fisher’s Exact Test; * Statistically significant (*p* < 0.05).

**Table 5:** Comparison between factors and occurrence of drug use problems, drug interactions, and other problems

**Factors**	**Drug use problems (n = 50) Frequency (Percentage, %)**			**Drug interactions (n = 63) Frequency (Percentage, %)**			**Other problems (n = 97) Frequency (Percentage, %)**		
	**Yes**	**No**	***p*-value**	**Yes**	**No**	*p***-value**	**Yes**	**No**	***p*-value**
**Elderly**									
Yes	17 (34.0)	68 (45.3)	0.215^a^	37 (58.7)	78 (56.9)	0.933^a^	57 (58.8)	58 (56.3)	0.836^a^
No	33 (66.0)	82 (54.7)		26 (41.3)	59 (43.1)		40 (41.2)	45 (43.7)	
**Polypharmacy**									
Yes	34 (68.0)	119 (79.3)	0.149^a^	58 (92.1)	95 (69.3)	0.001^a^*	74 (76.3)	79 (76.7)	> 0.999^a^
No	16 (32.0)	31 (20.7)		5 (7.9)	42 (30.7)		23 (23.7)	24 (23.3)	
**Duration of hospital stay**									
≤ 1 week	37 (74.0)	106 (70.7)	0.786^a^	44 (69.8)	99 (72.3)	0.854^a^	65 (67.0)	78 (75.7)	0.227^a^
> 1 week	13 (26.0)	44 (29.3)		19 (30.2)	38 (27.7)		32 (33.0)	25 (24.3)	
**Microvascular complications**									
Yes	31 (62.0)	93 (62.0)	> 0.999^a^	41 (65.1)	83 (60.6)	0.652^a^	65 (67.0)	59 (57.3)	0.204^a^
No	19 (38.0)	57 (38.0)		22 (34.9)	54 (39.4)		32 (33.0)	44 (42.7)	
**Cardiovascular disease**									
Yes	27 (54.0)	94 (62.7)	0.358^a^	53 (84.1)	68 (49.6)	< 0.001^a^*	63 (64.9)	58 (56.3)	0.270^a^
No	23 (46.0)	56 (37.3)		10 (15.9)	69 (50.4)		34 (35.1)	45 (43.7)	
**Renal impairment**									
Yes	22 (44.0)	78 (52.0)	0.414^a^	33 (52.4)	67 (48.9)	0.761^a^	49 (50.5)	51 (49.5)	> 0.999^a^
No	28 (56.0)	72 (48.0)		30 (47.6)	70 (51.1)		48 (49.5)	52 (50.5)	
**Liver impairement**									
Yes	4 (8.0)	7 (4.7)	0.472^b^	0 (0)	11 (8.0)	0.018^b^*	5 (5.2)	5 (5.8)	> 0.999^a^
No	46 (92.0)	143 (95.3)		63 (100)	126 (92.0)		92 (94.8)	97 (94.2)	
**Hyperlipidemia**									
Yes	17 (34.0)	68 (45.3)		37 (58.7)	78 (56.9)		57 (58.8)	58 (56.3)	0.836^a^
No	33 (66.0)	82 (54.7)	0.215^a^	26 (41.3)	59 (43.1)	0.933^a^	40 (41.2)	45 (43.7)	

^a^ Computed using Continuity Correction; ^b^ Computed using Fisher’s Exact Test; * Statistically significant (*p* < 0.05).

In addition, polypharmacy (*p* = 0.001) and cardiovascular events (*p* < 0.001) were found to be associated with drug interactions. Patients with polypharmacy or cardiovascular disease were more susceptible to potential drug interactions than those without these two factors. Moreover, a positive association between length of hospital stay and drug choice problem was discovered (*p* = 0.004). Those who stayed not more than one week had a higher likelihood of experiencing a drug choice problem.

## Discussion

The mean age of this study population was higher compared with published local data which evaluated the diabetes control of 1670 patients in Malaysia [3]. Both local and global data revealed that in developing countries like Malaysia, the prevalence of diabetes mellitus was highest in the age group between 45 and 64 years [2] but in this study only 36% of the patients fell into this age group. The higher mean age reported in this study might be because the sample was not representative of the whole population. It also suggests that more older hypertensive diabetic patients were admitted to the ward than were younger patients.

### Drug-related problems

In this study, there was an average of 1.9 ± 1.2 DRPs per patient. To date, there has been no comparable study done specifically on DRPs in T2DM patients with hypertension both locally and globally. The number of DRPs identified was only half the number detected by some other studies which were conducted in diabetes mellitus patients [10,11,25]. When compared with a recent study with an almost equivalent sample size (193 geriatric clinic patients in Taiwan), which also used the PCNE classification system, the average number of problems identified was 2.2 ± 1.6 per patient, slightly higher than in this study [21].

Although a similar PCNE classification of DRPs was used, the discrepancy with the study by Van Roozendaal *et al.* (2009) [11] could be due to the different methods and references used to identify DRPs. For example, the concurrent use of an ACE inhibitor and a sulfonylurea or insulin was considered as a potential DRP in that study and accounted for 46 cases out of the 682 DRPs detected. However, this combination of drugs was not considered as a potential drug interaction in this study because there is a lack of strong evidence of interaction [29]. Also, Van Roozendaal *et al.* (2009) [11] could not identify any possible contraindications as no information on patients’ renal and hepatic functions were successfully retrieved but this is not the case in this study as several contraindications were identified based on patients’ medical records and laboratory results.

Apart from that, the discrepancy with other studies may be attributed to the differences in study method and setting, different classification systems of DRPs used, and different methods to assess DRPs. Both the studies by Haugbolle & Sorensen (2006) [10] and Eichenberger *et al.* (2011) [25] conducted home visits and interviews. Also, the Problem Intervention Documentation (PI-Doc) coding system was used in the study by Haugbolle & Sorensen (2006)[10] whereas the classification system of DRPs used by Eichenberger *et al.* (2011)[25] was unclear, as it was not mentioned in its Methods section. Also, the clinical knowledge of the investigator(s) might also influence the assessment and identification of DRPs.

This study revealed that 90.5% of the patients had at least one DRP, which was much greater than the 80.7% reported by Haugbolle & Sorensen (2006) [10]. However, in a study conducted on ambulatory hemodialysis patients, 97.7% of the patients were found to have at least one DRP [32]. This variation across the studies can be attributed to the different study populations and study methods.

### Adverse reactions

In this study, almost one-third of the adverse drug reactions implicated antidiabetic drugs. Similar to the finding by Van Roozendaal & Krass (2008) [11], there was a potential risk of hypoglycemia in patients receiving oral antidiabetic drugs or insulin. Antihypertensive agents were also commonly associated with adverse reactions [33] and this finding was clearly demonstrated in this study. Calcium channel blockers caused a higher incidence of adverse reactions than diuretics, consistent with a study in an outpatient setting by Goncalves *et al.* (2007) [34]. Therefore, all these potential adverse reactions should be taken into consideration, especially in the elderly who might suffer significant deleterious effects. However, since this study was retrospective in nature, only the ones that were important for the hospital admission were noted.

### Drug choice problems

A drug choice problem was the second most common DRP in this study and this finding was comparable to other studies [10,11]. In this study, most of the contraindications identified were related to the use of metformin. Approximately 24% of the patients who received metformin were found to have contraindications. This was much less compared with the study carried out by Sweileh (2007) [35], in which up to 60% of the patients receiving metformin had contraindications to it. This difference is probably due to the variations in defining metformin’s contraindications. For instance, the decompensated stage, but no other stage of congestive heart failure, was defined as a contraindication in this study.

Apart from metformin, drugs that were categorized as high risk in the modified Beers criteria were frequently prescribed to the elderly, placing them at higher risk of developing drug toxicity [4,31]. The high frequency of drug choice problems may highlight a need for the health care providers to pay more attention when prescribing these drugs to older hypertensive diabetic patients.

### Dosing problems

In this study, excessive dosage was the most frequent dosing problem. Also, there were cases of subtherapeutic dosages and inappropriate durations of treatment. The percentage of dosing problems in this study was higher than that reported by Van Roozendaal and Krass (2009) [11] and this was probably due to the lack of assessment of patients’ renal and hepatic functions in the latter study.

Ranitidine was the most implicated drug for dosing problems. In clinical practice, dosage adjustments of ranitidine are not frequently applied although recommended by manufacturers and this is probably because its potential adverse effects are underestimated [36,37]. In a study by Manlucu *et al.* (2005) [37], H_2_-receptor antagonists were demonstrated to significantly increase the area under the curve (AUC) and the elimination half-life (*t*_1/2_) of serum drug concentrations when the glomerular filtration rate (GFR) was decreasing. Dosage reduction of drugs in patients with impaired renal function and low GFR may prevent adverse effects and decrease unnecessary drug expenditures [36,37]. Therefore, efforts should be made to minimize these dosing errors such as the involvement of a pharmacist in deciding the dosing of drugs or a computerized dosing program [37].

### Drug use problem

The most frequent drug use problem encountered was “drugs not taken by patients prior to admission”, which were mostly antihypertensive and antidiabetic drugs. In this study, forgetfulness might be one of the reasons for non-adherence. Also, complicated regimens for the treatment of diabetic complications may contribute to non-adherence. A systematic review confirmed the poor compliance in diabetic patients who were prescribed diabetic medications, whether oral agents or insulin [12]. Non-adherence has proven to be associated with negative outcomes such as higher A1c levels and blood lipid levels in diabetes patients [12,38].

### Drug interactions

In this study, the drugs that were most implicated in drug interactions were aspirin, clopidogrel, simvastatin and amlodipine. By contrast, beta-blockers, non-steroidal anti-inflammatory agents (NSAIDs) and ACE inhibitors were the drug most involved in drug interactions in a study conducted in Singapore [39]. The differences in prescribing patterns and practice in different hospitals may explain this discrepancy. The drug interactions identified in this study were mostly based on established literature and evidence. In clinical practice, several drugs can still be used together, yet close monitoring is fundamental and any toxicity should be identified and immediately followed by corrective actions.

### Others

Many of the patients in this study did not engage in regular physical activity, did not adhere to diabetic diets, did not perform any routine blood glucose monitoring, and defaulted on follow-up or medications, and all these problems were clearly stated by the health care providers in the medical records. These problems would lead to poor glycemic control and accelerate the development or worsening of diabetic complications [40].

A local study conducted in an endocrine clinic in a teaching hospital in Kuala Lumpur identified barriers to optimal control of Malay type 2 diabetic patients by interviewing 18 patients and health care providers. It was not surprising to find that most of the patients had a lack of understanding of the disease itself and of its management, which would eventually contribute to non-adherence [41]. This is particularly true for the elderly as they tend to have decreased memory, health beliefs not in line with drug therapy, and often neglect the importance of adherence to medications and dietary control. Hence, counseling may be important to increase the awareness and knowledge of this patient population since they frequently encounter these problems.

### Causes of DRPs

The results from our study revealed that among all the causes, “drug or dose selection” was the most frequently identified cause for DRPs such as drug choice problems, dosing problems or drug interactions. According to the PCNE classification of DRPs, this domain of causes is directly related to the drug or dosage selection while the other domains are concerned with patient-related causes [22].

On the other hand, patients usually had “drug use problems” caused by “drug use process, lack of information, and physiological or patient factor”. Generally, the number of causes identified was lower than the causes identified in other studies such as Chan *et al.* (2011) [21]. This is because most of the problems identified were matched with the one most relevant cause rather than several causes, which might be seen in other studies.

### Factors found to be associated with DRPs

Generally, this study did not identify any factors with a statistically significant association with DRP. This was in agreement with a study by Koh *et al.* (2005) [39] which found no statistical correlation between DRPs and age or gender. Research by Samoy *et al.* (2006) [42] also concluded that there were no risk factors for drug-related hospitalization in a tertiary care hospital in Canada. One of the possible explanations is the nature of the PCNE classification system (6 problem domains with 22 categories) which could possibly affect the results. Also, it remains unclear whether the result was affected by the sample size (Samoy *et al.*, 2006) [42].

When examining each of the problem domains with several possible factors, statistically significant associations were observed. These associations should receive the attention of the health care providers in order to minimize preventable DRPs.

### Elderly

In our study, the non-elderly were found to be associated with drug choice problems. From the literature reviewed, the findings on the association between age and DRPs are conflicting. In one study on the elderly in an ambulatory setting, age of 80 and above was found to be an independent risk factor for adverse drug events [43]. While a study by Chan *et al.* (2011) [21] on geriatrics also reported a significant association between age and DRPs, a few studies did not agree with this finding. A study on hospitalized patients from several internal medicine departments found that age was not a risk factor of DRPs [15]. Similarly, Koh *et al.* (2005) [39] did not report any statistically significant correlation between these two.

### Polypharmacy

It is a well-known fact that polypharmacy is strongly associated with DRPs and this has been shown by numerous studies [12,14,15,43]. It has been reported that a one unit increase in the number of drugs can lead to an increase of 8.6% in the number of DRPs [43].

The results of our study revealed a significant statistical association between polypharmacy and drug interactions, which was consistent with the result from Moura *et al.* (2009) [44], a retrospective study on drug interactions in a public hospital in Brazil. The increasing number of drugs used can lead to an increased risk of potential drug interactions [23,45]. Since polypharmacy is an inherent problem in T2DM patients with hypertension, the clinically important and significant drug interactions should be routinely checked and monitored [9].

### Renal impairment

Renal impairment was associated with both the drug choice problem (*p* = 0.029) and the dosing problem (*p* = 0.027). Drugs with doses that were higher than required were often prescribed to T2DM patients with hypertension and renal impairment in our study. Also, dosage adjustment was commonly ignored by physicians, suggesting that the severity of inappropriate drug and dosing selection might be underestimated [36,37]. DRPs were common among patients with renal impairment due to co-existing medical conditions, as most of them were receiving multiple medications which require dosage adjustment and routine monitoring [36].

The study by Manley *et al.* (2003) [32] revealed that in ambulatory hemodialysis patients, the presence of diabetes mellitus is one of the factors associated with DRPs. In other words, diabetes patients on hemodialysis were more likely to experience DRPs. Similarly, Leendertse *el al*. (2008) [14] also found that impaired renal function was a risk factor for potentially preventable DRPs. Therefore, in patients with renal impairment, dosage adjustment and close monitoring of renal function are fundamental in order to minimize drug toxicity or subtherapeutic effect [46].

### Cardiovascular events

Patients with cardiovascular events had more potential drug interactions than patients without cardiovascular disease in our study. This can be explained by the wide use of cardiovascular drugs such as antihypertensive drugs, antiplatelet drugs, anticoagulants, and lipid lowering drugs in T2DM patients with hypertension. Many studies concluded that the most common drug category involved in DRPs was cardiovascular agents [12,47]. Also, cardiovascular events often add an additional burden to patient conditions and complicate their therapies.

### Duration of stay

Patients who stayed for not more than one week in the hospital tended to experience the drug choice problem as compared with those who stayed for more than one week. This finding of our study was not in line with that reported by Moura *et al.* (2009) [44], which revealed an association between duration of hospital stay and potential drug interactions.

Another study on hospitalized cancer patients also found a correlation between duration of hospital stay (≥ 6 days) and potential interactions [48]. The relationship between length of hospital stay and DRPs needs to be investigated in future studies since there is a lack of published literature investigating this association.

### Study limitations

Because of the retrospective nature of our study, the identification and assessment of the DRPs were based on the data available from the medical records with reference to established literature and guidelines. The number of studied patients was less than 50% of potentially eligible patients.

## Conclusions

The most common drugs that were used in T2DM patients with hypertension were amlodipine, insulin, aspirin and simvastatin. Polypharmacy and multiple comorbidities were common in this patient population.

The most common DRPs were “others” (i.e., insufficient awareness of health and diseases), drug choice problems, dosing problems, and drug interactions. The most implicated drugs were aspirin, clopidogrel, simvastatin, amlodipine and metformin.

Several factors were found to have statistically significant associations with the six domains of DRPs, including renal impairment, polypharmacy, cardiovascular disease, elderly age, and duration of hospital stay.

Special attention should be given to T2DM patients with hypertension and risk factors who are prescribed commonly implicated drugs.

## Competing interest

The authors have no conflict of interest to report.

## Authors' contributions

HZH has made substantial contributions to conception and design, acquisition of data, analysis and interpretation of data and drafting the manuscript or revising it critically for important intellectual content. HFW has been involved in acquisition of data and analysis and interpretation of data. HZH and HFW have given final approval to the version to be published. Both authors read and approved the final manuscript.

## Pre-publication history

The pre-publication history for this paper can be accessed here:

http://www.biomedcentral.com/1472-6823/13/2/prepub
